# Age-Related Immune Profile of the T Cell Receptor Repertoire, Thymic Recent Output Function, and miRNAs

**DOI:** 10.1155/2020/5910823

**Published:** 2020-12-02

**Authors:** Yan Xu, Ling Xu, Cunte Chen, Yikai Zhang, Chengwu Zeng, Zhenyi Jin, Shaohua Chen, Bo Li, Xianfeng Zha, Zhinan Yin, Yangqiu Li

**Affiliations:** ^1^Key Laboratory for Regenerative Medicine of Ministry of Education, Institute of Hematology, School of Medicine, Jinan University, Guangzhou 510632, China; ^2^The First Affiliated Hospital, Jinan University, Guangzhou, 510632 Guangdong, China; ^3^The Biomedical Translational Research Institute, Jinan University, Guangzhou, 510632 Guangdong, China; ^4^Department of Clinical Laboratory, First Affiliated Hospital, Jinan University, Guangzhou 510632, China

## Abstract

**Background:**

T cell immunity plays a central role in the body's defense system, including maintaining homeostasis and preventing tumorigenesis and viral infection. Immune system functions degenerate with age, leading to immune senescence. Physiologically, immune senescence is characterized by a decrease in T cell receptor diversity, naive T cell deficiency, and alterations in T cell immune-related miRNAs. However, little is known about the characteristics of T cell immunosenescence in Chinese individuals.

**Results:**

A significant decrease in the miR-17, miR-92a, and miR-181a levels in PBMCs was detected with age. The miR-92a and miR-181a levels were upregulated in CBMCs when comparing healthy individuals to group I (0~9 years), whereas miR-17 was downregulated. The sjTREC level in PBMCs was negatively correlated with age, and a sharp decrease in sjTRECs was found between groups I and II (10~19 years). Twenty-four TCR V*β* subfamilies could be detected in most samples, and most displayed polyclonality, while skewed expression of the V*β* subfamilies as well as an increased oligoclonal tendency was found with age. Similarly, the frequencies of the TCR V*γ* and V*δ* subfamilies decreased with age, and the alteration in clonality appeared to be stable at different ages.

**Conclusion:**

We made the novel observation of T cell immunosenescence with age in Chinese individuals, which may provide information for immune targets to enhance the T cell immune response in immunotherapy settings for elderly patients.

## 1. Introduction

Aging leads to numerous alterations in physiological systems, and the immune system is one of the most influential systems resulting in immunosenescence. In particular, T cell subfamilies are associated with a reduced capacity for immune surveillance and increased cancer incidence [1, 2]. Physiologically, T cell immunosenescence is characterized by reduced T cell regeneration, lower differentiation ability [3], decreased naive T cell numbers [4], lesser T cell receptor (TCR) diversity [5], low activation, failure to respond to vaccines and neoantigens [6], and increased T cell terminal differentiation [7, 8].

Recently, investigators have been seeking biomarkers for immunosenescence with the hopes of enhancing the T cell response to vaccines and improving immunotherapy for cancer [9]. Several characteristics of T cell aging have been reported, such as loss of the costimulatory molecule CD28 [10]; high expression of Fas/Fas ligand on T cells [11]; decreased IL-2 and IFN-*γ*; increased IL-4, IL-6, IL-10, and TNF-*α* secretion [12]; decreased naive T cells; and increased memory T cells [13]. Based on the immunocompetence of T cells toward antigens and maintaining immune homeostasis, thymic recent output function and TCR repertoire diversity are the essential indices for evaluating T cell immunity. For example, low naive T cells and a skewed TCR repertoire result in poor T cell immune recovery after chemotherapy and hematopoietic stem cell transplantation [14–16].

Evaluation of TCR diversity and T cell clonality is based on analysis of the length and sequence of the complementarity-determining region 3 (CDR3) resulting from TCR V(D)J gene rearrangement [14, 17, 18]. Typical methods for evaluating thymic output function are based on quantitative analysis of signal joint T cell receptor excision circles (sjTRECs). sjTRECs are circular excision products formed by TCR gene segment deletion during TCR rearrangement steps. These molecules are stable, cannot amplify, are exported from the thymus to the periphery with thymic recent emigrants, and are ultimately diluted during T cell proliferation. Since 1998, sjTRECs have been used as markers for T cell neogenesis to represent the number of naive T cells as well as thymic output function [19, 20].

Recently, increasing data have shown that epigenetic regulation is an important factor in aging. It has been reported that microRNAs (miRNAs), such as the miR-17-92 cluster, miR-24, miR-92a, miR-103, miR-107, miR-128, miR-130a, miR-132, miR-142-3p/5p, miR-146a/b, miR-155, miR-221, miR-223, miR-496, and miR-1538 may be involved in cellular senescence and aging [21, 22]. miR-17-92 and miR-181a have been reported to play a critical role in T cell differentiation and proliferation [23, 24]. Thus, in this study, we analyzed the expression of miR-17, miR-92a, and miR-181a with aging combined with changes in TCR diversity and sjTRECs to attempt to characterize the potential role of the immune profile as biomarkers for immunosenescence in the Chinese population.

## 2. Materials and Methods

### 2.1. Samples

Cord blood was obtained from 20 full-term healthy babies at delivery. Peripheral blood samples were collected from 160 healthy donors including 85 males and 75 females with a median age of 40 years (range: 0 to 70 years) (Table 1). The healthy donors had a healthy status without any type of cancer, type 2 diabetes, or autoimmune disease. All experiments were conducted with the understanding and consent of each participant, and ethical approval was obtained from the Ethics Committee of the First Affiliated Hospital, School of Medicine, Jinan University.

Cord and peripheral blood mononuclear cells (CBMCs and PBMCs) were isolated, DNA and total RNA were extracted, and cDNA for miR-17, miR-92a, and miR-181a was reverse-transcribed using the miScript II RT Kit (QIAGEN, Duesseldorf, Germany). First-strand cDNA for target gene assays was reverse-transcribed using random hexamer primers and the High Capacity cDNA Reverse Transcription kit (ABI, Carlsbad, CA, USA) according to the manufacturer's instructions.

### 2.2. Quantitative Real-Time RT-PCR (qRT-PCR) for miRNA Detection

The expression levels of miR-17, miR-92a, and miR-181 were determined using the primers in Table 2 and the miScript SYBR Green PCR kit (QIAGEN, Duesseldorf, Germany). The miRNA expression levels were normalized to RNU6B snRNA [25].

### 2.3. Real-Time Quantitative PCR for sjTREC Detection

Quantitative detection of *δ*Rec-*ψ*J*α* sjTRECs in genomic DNA from CBMCs and PBMCs was performed by TaqMan real-time PCR as described previously [26].

### 2.4. RT-PCR and GeneScan Analysis for the Expression and Clonality of the TCR V*β*, TCR V*γ*, and TCR V*δ* Subfamilies

The expression of the TCR V*β*, TCR V*γ*, and TCR V*δ* subfamilies was detected by RT-PCR using 24 V*β*, 3 V*γ*, and 8 V*δ* gene primers and a single C*β*, C*γ*, or C*δ* primer, respectively, for the first run (unlabeled PCR for amplification). Subsequently, runoff PCR was performed using fluorophore-labeled primers (C*β*-FAM, C*γ*-FAM, or C*δ*-FAM), and the products were the analyzed for clonality by GeneScan analysis. PCR and GeneScan analyses were performed as previously described [27–29].

### 2.5. Statistical Analyses

Pearson correlation and linear regression analysis was used to estimate the correlation between the expression level of miR-17, miR-92a, miR-181a, and TRECs and age from all the groups. The differences in miR-17, miR-92a, miR-181a, and TRECs among different age groups were analyzed by one-way ANOVA. Results are expressed as mean ± SEM. All data analyses, including statistical calculations and graphical displays, were performed using SPSS 13.0 and GraphPad software. The readxl and circlize packages in R, version 3.6.1 (http://www.r-project.org/), were used for constructing chord diagrams.

## 3. Results

### 3.1. Downregulation of miR-17, miR-92a, and miR-181a with Aging

**Figure 1:**
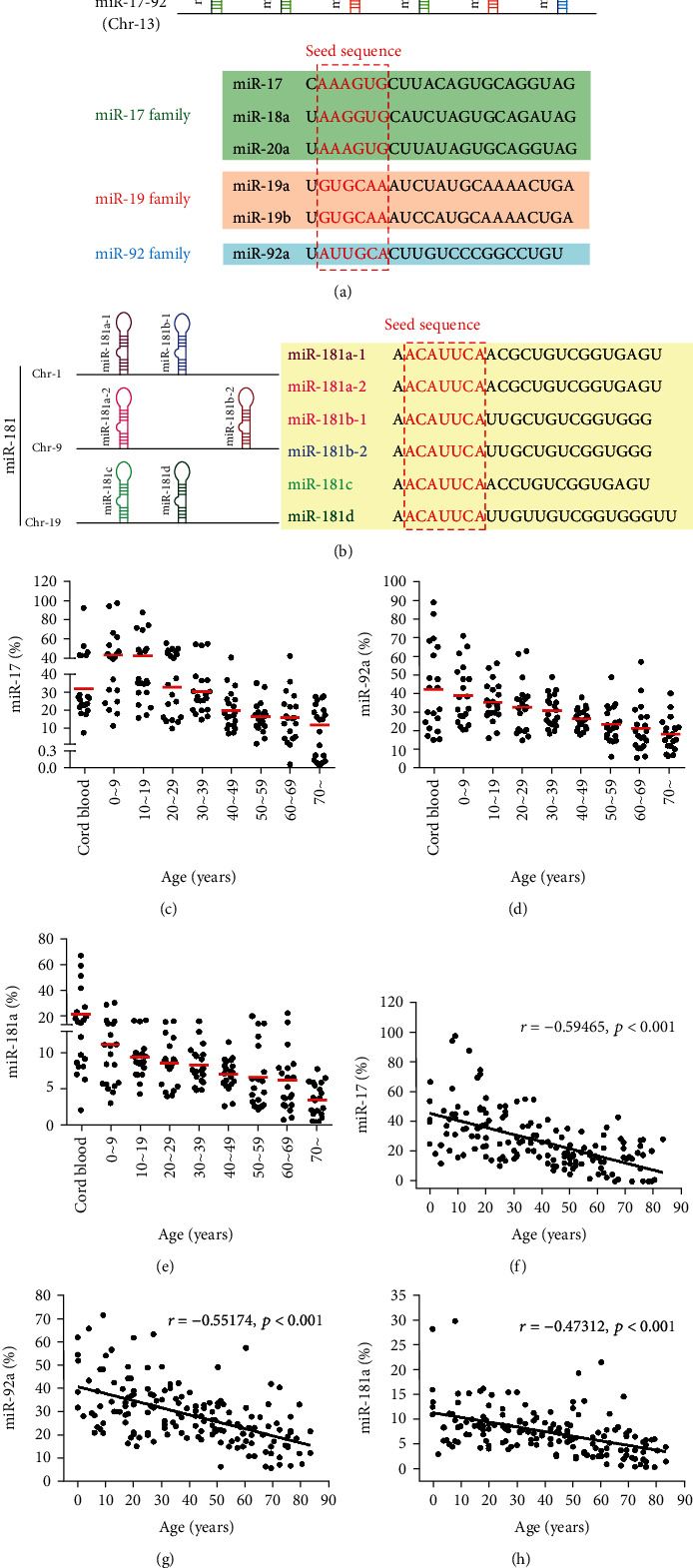
The expression levels of miR-17, miR-92a, and miR-181a in PBMCs are downregulated with age. (a) The location and members of the miR-17-92 cluster and the sequences of the six members in three families. Green: members of the miR-17 family; red: members of the miR-19 family; blue: members of the miR-92 family. (b) miR-181 family members and their genomic locations. (c–e) Quantification of the data revealed that the expression levels of miR-17 (c), miR-92a (d), and miR-181a (e) were downregulated with age (*n* = 20/each age group). (f–h) A negative correlation between age and miR-17 (f), miR-92a (g), and miR-181a (h) is shown.

The expression levels of miR-17, miR-92a, and miR-181a were quantitatively detected in peripheral blood mononuclear cells (PBMCs) from 160 healthy individuals (HIs) of different ages (0 to 70 years, groups I to VIII) and cord blood mononuclear cells (CBMCs) from 20 cases. The expression of three miRNAs in PBMCs was found to be significantly downregulated with age. Moreover, compared to group I (0~9 years), we found a lower expression of miR-17 (*p* < 0.0001) in group V (40~49 years), group VI (50~59 years), group VII (60~69 years), and group VIII (over 70 years), and the sharpest declines were found for those between the ages of 30 and 40 followed by those between 10 and 20. However, there was no statistically significant difference between neighboring age groups. The miR-17 level in the CBMC group was lower than that in group I, group II (10~19 years), and group III (20~29 years) and higher than that in groups IV (30~39 years) to VIII. Statistical significance was found when comparing CBMCs in groups VII and VIII (*p* < 0.05) (Figure 1(c)). Because miR-17 and miR-92a belong to the same gene cluster (Figure 1(a)), we found that they had similar expression characteristics. For example, compared with group I, low miR-92a expression was found in groups VI through VIII (*p* < 0.01), and the sharpest decline in the miR-92a level was also found for those between 30 and 40 years old. However, unlike miR-17, the expression of miR-92a also had its own characteristics. miR-92a was upregulated in CBMCs when compared with all groups of PBMCs, and statistical significance was found for CBMCs when comparing groups V to VIII (*p* < 0.05) (Figure 1(d)). For miR-181a (Figure 1(b)), when compared with group I, a significant lower expression was found in group VIII (*p* < 0.01). The sharpest decline was found between 60 and 70 years followed by 0 and 10 years. Moreover, miR-181a was highly expressed in CBMCs in comparison with all PBMCs (Figure 1(e)). Furthermore, the expression levels of miR-17, miR-92a, and miR-181a had a significant negative correlation with aging (*r* = −0.59465, -0.55174, and -0.47312, all *p* < 0.0001) (Figures 1(f)–1(h)).

### 3.2. Thymic Output Function Progressively Declines during Aging

**Figure 2:**
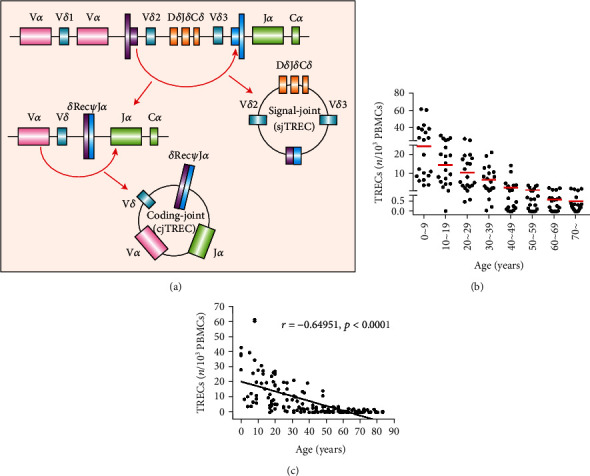
sjTREC are decreased in PBMCs with age. (a) Schematic diagram of T cell receptor excision circle (TREC) formation. TRECs are pieces of DNA fragments generated during TCR gene rearrangement in the thymus that are exported from the thymus to the periphery in T cell episomes. TRECs are not duplicated during mitosis and remain stable for a long time. However, as T cells divide, the level of TRECs is gradually diluted. Generation of TRECs occur in >70% of all new (naive) T cells, and they can be detected by PCR. (b) TREC levels in PBMCs from 160 healthy individuals at different ages (0 to 70 years old). (c) An inverse correlation between the TREC count and donor age was found.

To characterize alterations in thymic output function over the lifespan, absolute quantitative analysis of the sjTREC (TREC) level in DNA samples from PBMCs and CBMCs was performed with 160 healthy individuals (HI) of different ages (0 to 70 years old) and 20 cord blood samples (Figure 2(a)) [20]. Significantly, the number of TRECs progressively declined with age, and the biggest drop was between the ages of 0 and 9 (Figure 2(b)). A strong negative correlation between the number of TRECs in PBMCs and age was found (*r* = −0.64951; *p* < 0.0001) (Figure 2(c)). Interestingly, there were no obvious alterations in the TREC level in the 50 to 59 age group (group VI), and a relatively lower number of TRECs was found for those ≥ 60 years. However, minimal thymic output function remained in elder individuals.

### 3.3. Skewed Distribution and Clonality of TCR V*β*, V*γ*, and V*δ* T Cells with Aging

**Figure 3:**
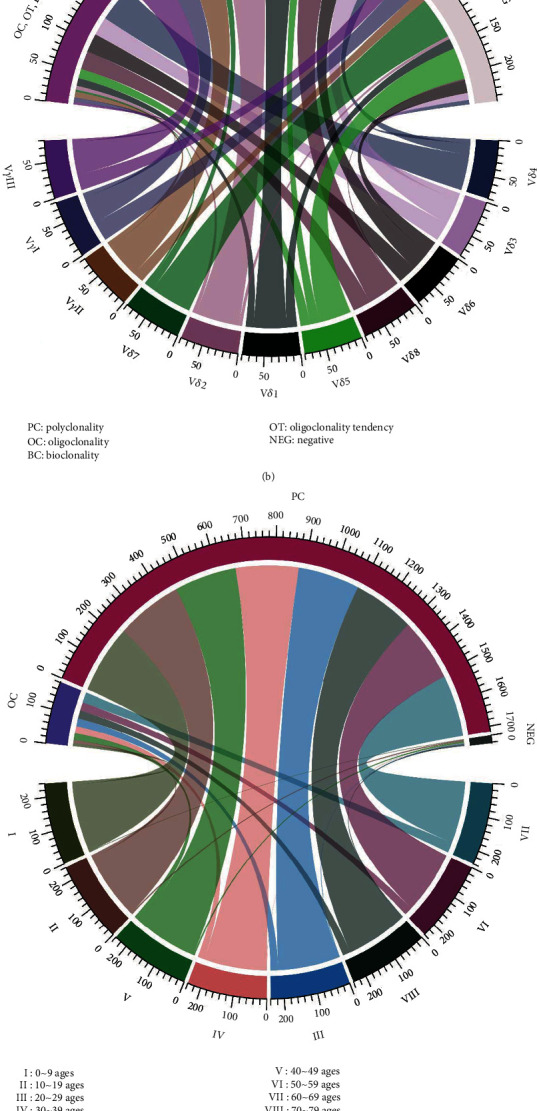
Chord diagram displaying the frequencies and clonalities of the TCR V*β*, V*γ*, and V*δ* subfamilies in different age groups. (a, b) Each sector of the circle represents one node of the TCR subfamilies, and its width indicates the expression frequencies of TCR V*β* (a), V*γ*, and V*δ* (b) that connect the clonalities of the TCR subfamilies. (c, d) Circularly arranged sectors represent the different age groups and the clonalities of the TCR V*β* (c), V*γ*, and V*δ* (d), and the scale bars indicate their relative expression frequencies (*n* = 10 in each age group).

**Figure 4:**
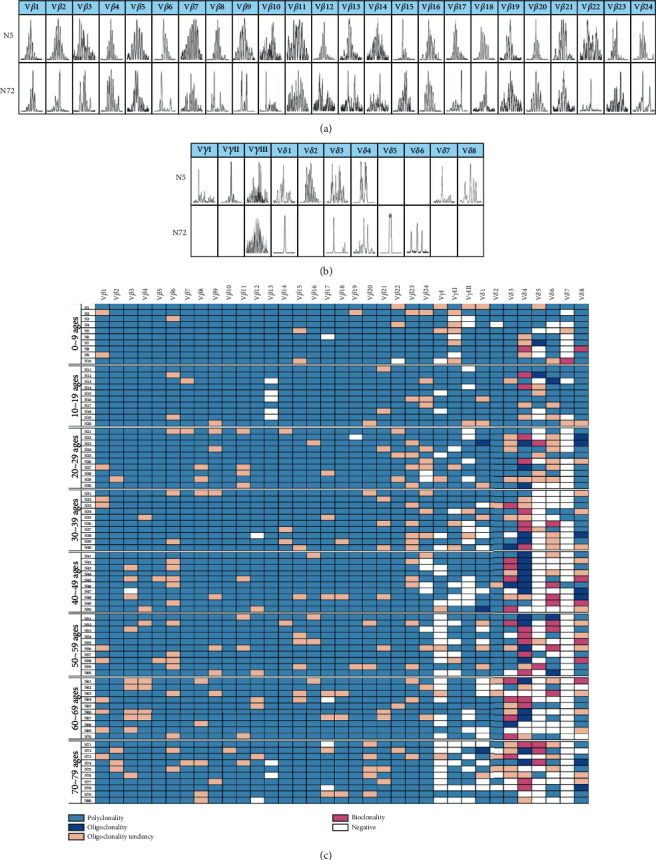
Distribution and clonality of the TCR V*β*, V*γ*, and V*δ* subfamilies in different age groups. (a) GeneScan analysis results from the TCR V*β*, V*γ*, and V*δ* subfamilies in PBMCs from two cases, group I (N5) and group VIII (N72). (b) The characteristic distribution and clonality of the TCR V*β*, V*γ*, and V*δ* subfamilies in PBMCs from 80 cases of healthy adult individuals.

TCR diversity and clonality are indices for T cell generation, proliferation potential, and the ability of specific amplifications to respond to antigen stimulation. In this study, we detected the distribution and clonality of the TCR V*β*, V*γ*, and V*δ* subfamilies in 80 cases in groups I to VIII using RT-PCR and GeneScan. First, the expression of TCR V*β*1, V*β*2, V*β*4~V*β*11, V*β*14~V*β*16, V*β*18, V*β*20, V*β*21, and V*β*23 could be detected in all samples (100%). The expression of V*β*3 (98.75%), V*β*12 (97.5%), V*β*17 (95%), V*β*19 (98.75%), and V*β*22 (98.75%) was absent in some cases, and the expression of V*β*13 and V*β*24 was lowest (91.25%) (Figure 3(a)). Based on the different CDR3 TCR rearrangement lengths, the clonality of the T cells was characterized as multipeaks, oligopeaks, and monopeaks corresponding to polyclonality, oligoclonality, and monoclonality, respectively, by GeneScan analysis (Figure 4(a)) [30]. V*β* polyclonality was identified in most samples, and oligoclonality was found for V*β*6 and V*β*23 in 23.75% of the samples. There was decreasing V*β* subfamily expression frequency with an increased oligoclonal tendency for V*β* T cells with age (Figures 3(c) and 4(c)). Second, for the V*γ* subfamily, V*γ*II was the main subfamily detected at 78.75% followed by V*γ*III at 70% and V*γ*I at 61.25% in 80 total PBMC samples (Figure 3(b)). Similarly, the V*γ* frequency decreased with age. Interestingly, an oligoclonal tendency was mainly found in group I, but there was no significant correlation with age (Figures 3(c), 3(d), 4(b), and 4(c)).

Finally, unlike the high expression frequency of the V*β* subfamilies in healthy individuals, the expression frequency of the V*δ* subfamilies was relatively different. The highest expression frequency was found for V*δ*8 at 97.5% followed by V*δ*2 at 93.75% and V*δ*4 at 91.25%. V*δ*5 and V*δ*7 expression was detected in only 37.5% and 18.75% of the samples, and greater than 20% of the V*δ* subfamilies was absent in groups IV to VIII, particularly in group VII (up to 36%) (Figures 3(b) and 3(c)). In addition, oligoclonality was relatively high for V*δ*4 (70%), and oligoclonal expansion increased significantly beginning with group III (Figures 3(c), 4(b), and 4(c)).

## 4. Discussion

Understanding T cell immune alterations in healthy individuals during aging may help us evaluate the immune status of patients with different diseases, particularly cancer patients who may receive immunotherapy. There are limited basic data regarding the T cell immune status in the Chinese population [7, 8]. In this study, we examined T cell receptor diversity, thymic recent output function, and expression of the T cell immune-related miRNAs miR-17, miR-92a, and miR-181a in healthy individuals of different ages ranging from 0 to over 70.

Several studies have reported a number of miRNAs mediating cellular senescence [31]; however, how these miRNAs contribute to the aging process remains unclear. miR-17 and miR-92a downregulation was found in human CD8^+^ T cells, while miR-181a declined with age in CD4^+^ T cells [32, 33]. In this study, we detected a change in miR-17, miR-92a, and miR-181a in healthy individuals ranging in age from 0 to over 70 years. Significantly, the expression of these miRNAs decreased with age, and we also characterized a declining tendency with age for the different miRNAs. Healthy individuals were divided into eight different age groups in intervals of ten years according to calendar age. In this manner, we could identify more detailed miRNA expression patterns with obvious variation trends. Interestingly, the miR-17 level was high in groups I to III (0 to 29 years) compared with that in the CBMC groups, while the expression levels of miR-92a and miR-181a were highest in CBMCs compared with all of the PBMC groups. The sharpest decline in the expression of miR-17 and miR-92a was between 30 and 40 years, and the sharpest decline in the miR-181a level was delayed to over 60 years. Overall, these novel results are in agreement with other reports demonstrating that miR-17, miR-92a, and miR-181a are downregulated in various tissues with age [34, 35]. Moreover, we found that the regulation of miR-17, miR-92a, and miR-181a may have different patterns during aging. Thus, we assume that ages 40 and 70 may be cutoff points for miR-17~92 and miR-181a aging, respectively. It has been reported that miR-17~92 targets the control of BIM and PTEN, and the decrease in miR-17 and miR-92a may result in inhibition of AKT-mTOR by increasing PTEN. mTOR is a well-known regulator of cell aging and the cell cycle [36]. Additionally, the mechanism underlying miR-181a regulation of aging is thought to be via inhibition of Bcl-2 and an increase in the proapoptosis genes caspase 9 and caspase 3 and cell death [37]. Further investigation of the relationship between miR-17 and miR-92a and the AKT-mTOR pathway and that between miR-181a and the Bcl-2 pathway may confirm their role in aging.

In this study, we also examined the expression profile of miR-17, miR-92a, and miR-181a in cord blood as it is known that cord blood contains greater numbers of naive T cells [38]. As expected, the miR-92a and miR-181a levels were higher than that in all groups of healthy individuals, and only the miR-17 level was lower than that in group I (0~9 years), suggesting that the expression of these miRNAs may be out of sync in cord blood, and the underlying explanation for this phenomenon remains to be investigated.

The sjTREC number in PBMCs or T cell subsets was used as a specific index for thymic recent emigrants and naive T cell number, which is important for evaluating T cell immunity [20]; however, little is known about how the numbers change with aging in the Chinese population. Thus, in this study, we quantified sjTRECs in PBMCs and CBMCs to determine baseline level data and plotted the changes in sjTREC numbers with age. We found that in 160 healthy volunteers of different ages, TRECs ranged from 0.004 to 95.124 copies/10^3^ PBMCs, and linear correlation analysis showed that the level of TRECs was negatively correlated with the ages. The thymus produces significantly less TRECs with increasing age, which is consistent with the results of foreign reports [39, 40].TREC levels were high up to age 10, then gradually declined with age, and the levels of TRECs decreased significantly after age 50, but they were still detectable above age 70 with limited amounts. Furthermore, the study by Pido-Lopez et al. pointed out that the TREC content in PBMCs is quadratic with age, and the best fit curve is *y* = 0.002*x*^2^ − 0.2139*x* + 7.96181, *R*^2^ = 0.601. The authors noted that TREC levels decrease with age at a rate of approximately 2% per year up to age 40 and at a rate of approximately 1.5% per year after age 40 [40]. In addition, the study by Naylor et al. also suggests that TREC levels decrease significantly after age 65. Before age 65, only a small fraction of new thymocytes are required to maintain diversity, but after age 65, this diversity is maintained through increased spontaneous T cell activation and the conversion of naive cells to memory cells. Additional studies have also confirmed that the age of 40-50 years may be a watershed in thymic output function. The two biggest drops in number were between 10 and 60 years [41]. Since 1998, sjTRECs have been used to evaluate the recovery of the thymus after treatment in HIV-infected and AIDS patients [19]. Decreased immune function and imbalance in the elderly increases susceptibility to infection and ultimately reduces their quality of life. Therefore, restoration of immune function is beneficial in maintaining the health of the elderly. We hypothesized that greater thymic output would lead to a larger pool of naive T cells to reproduce a more competent immune response and reduced mortality.

The TCR includes *α*, *β*, *γ*, and *δ* chains, which are functionally expressed on the T cell surface as either *α*/*β* or *γ*/*δ* heterodimers corresponding to *αβ*+ or *γδ*+ T cells [42]. Each functional TCR gene is encoded by TCR variable (V), diversity (D, only for *β* and *δ* chain), and joining (J) segments in which the length and sequence of the complementarity-determining region 3 (CDR3) is specific for each TCR rearrangement, which could be used as a biomarker for evaluating the TCR repertoire diversity [43, 44]. In this study, we analyzed the CDR3 of the TCR V*β*, V*γ*, and V*δ* subfamilies to characterize the diversity and clonality of the *αβ*+ and *γδ*+ T cells with aging. For the TCR V*β* subfamilies, the frequency appeared to decrease slightly with age, while an increasing oligoclonality of TCR V*β* T cells with age also indicated reduced TCR diversity in the same subfamilies. Thus, evaluation of TCR diversity may include changes in both TCR V*β* frequency and clonality. Moreover, we recently found that T cell exhaustion displays a different distribution of TCR V*β* subfamily T cells [17]. Thus, it may be worth combining the detection of T cell exhaustion and senescence with aging. The expression pattern of the TCR V*γ* subfamilies appeared to be similar to that of V*β*, while for the TCR V*δ* subfamilies, the distribution and clonality were different from the TCR V*β* subfamilies with aging. High expression was only found for V*δ*2, V*δ*4, and V*δ*8. Oligoclonality was relatively high, particularly for V*δ*4, and significantly, the expression of the V*δ* subfamilies was reduced over age 30. There are reports that V*γ*9/V*δ*2 (V*γ*II/V*δ*2) T cells, which have cytotoxic effects on tumor or infected cells, are the major population of *γδ* T cells in peripheral blood in healthy individuals [45]. In this study, we also found that V*γ*II/V*δ*2 had the highest subfamily expression, but we also found that the V*γ*II/V*δ*8 subfamilies also had a high frequency of expression in this group of samples. In a previous study, our group demonstrated that the TCR V*δ* gene is mainly comprised of V*δ*1, V*δ*2, V*δ*3, and V*δ*8 chains. In addition, V*δ*8 was the most frequently clonally expanded T cell subfamily member in patients with refractory anemia with an excess of blasts and acute myeloid leukemia [30, 46]. However, the function of V*δ*8^+^ T cells is not clear. For this phenomenon, further analysis is needed to determine whether it is a characteristic of the TCR lineage in the Chinese population.

We know that most common aging-associated diseases are cardiovascular disease, cancer, arthritis, dementia, cataract, osteoporosis, diabetes, hypertension, and neurodegenerative diseases such as Alzheimer's disease, Huntington's disease, Parkinson's disease, and amyotrophic lateral sclerosis. Research on immunosenescence and testing methods can provide a basis for diagnosis and further understanding of immunodeficiency-related diseases and provide a basis for seeking specific therapies.

## 5. Conclusions

In conclusion, in this study, we reported for the first time the characteristics of T cell immunity based on thymic recent output function, TCR diversity, and T cell-related miRNAs in the healthy Chinese population; we made the novel observation of T cell immunosenescence with age. The biggest drops of sjTREC number clearly point to the age-related timing change of thymic function in Chinese individuals; combining with the dynamic change of miR-92a, miR-181a, and miR-17 levels, it may indicate the regulating time points in T cell proliferation with age. Finally, the findings of the distribution pattern of reduced TCR diversity with increasing oligoclonality of TCR subfamily T cells with age may help to build a foundation for immunosenescence evaluation indices and provide information for immune targets for enhancing the T cell immune response in the immunotherapy setting in elderly patients.

## Figures and Tables

**Table 1:** Sample demographic information.

Groups (years)	Male (*n*)	Female (*n*)	Median age (years)
0~9	9	11	6
10~19	9	11	17
20~29	6	14	25
30~39	9	11	34.5
40~49	8	12	45
50~59	12	8	53
60~69	10	10	64
≥70	12	8	75.5
CMBCs	10	10	0

**Table 2:** miRNA primer sequences.

Primer	Sequence (5′→3′)
*miR-17-3p*	ACUGCAGUGAAGGCACUUGUAG
*miR-92a*	UAUUGCACUUGUCCCGGCCUGU
*miR-181a-5p*	AACAUUCAACGCUGUCGGUGAGU
*RNU6-2*	ACGCAAATTCGTGAAGCGTT

## Data Availability

The datasets used and/or analyzed during the current study are available from the corresponding author on reasonable request.
